# COVID-19-Induced Downsizing and Survivors’ Syndrome: The Moderating Role of Transformational Leadership

**DOI:** 10.3389/fpsyg.2022.833116

**Published:** 2022-04-08

**Authors:** Farah Samreen, Sadaf Nagi, Rabia Naseem, Habib Gul

**Affiliations:** ^1^Institute of Business and Management, University of Engineering and Technology, Lahore, Pakistan; ^2^Department of Business Administration, Federal Urdu University of Arts, Sciences and Technology, Islamabad, Pakistan; ^3^School of Graduate Studies MBA Department, Kardan University, Kabul, Afghanistan

**Keywords:** COVID-19, organizational identification, job uncertainty stress, transformational leadership, affective commitment, survivor’s syndrome

## Abstract

Downsizing due to COVID-19 (COV-DS) and its consequences on laid-off employees has attracted the attention of many researchers, around the globe. However, the underlying mechanisms that explain the effects of COVID-19 downsizing (COV-DS) on the employees who have survived cutoffs remain underexplored. Grounded in the conservation of resources theory, this manuscript aims to study the causal path through which COV-DS reduces the survivors’ affective commitment. The current study proposes the mediation of survivors’ job uncertainty, stress, and organizational identification between COV-DS and survivors’ affective commitment. This study also posits the moderating role of transformational leadership between COV-DS and both the mediators. The extant study has employed WARPED partial least square WARP PLS 7 and Hayes Process Macro to test the hypothesized relationships. Using the sample of 274 employees from the private sector of Pakistan, it was found that job uncertainty’s stress strongly mediates the relationship between COV-DS and survivors’ affective commitment. While mediation of survivors’ organizational identification was not proven to be significant. However, with the moderation of transformational leadership, both the mediators were proven to be significant.

## Introduction

The global health crisis of the COVID-19 has presented unprecedented difficulty for organizations and people working in them and labeled as a “job-killer.” Hundreds of thousands are compelled to shut down their businesses and millions are laid off. This situation has an immense adverse impact on employees’ psychological and physical health (Blustein and Guarino, 2020; Lim et al., 2020; Nicola et al., 2020; Islam et al., 2021; Tu et al., 2021). Hence, there arises a compelling need to study the phenomenon of downsizing that occurred as a consequence of COVID-19 and its effect on the organizational employees (Tu et al., 2021; Khokhar et al., 2022). Though the downsizing phenomenon is not new to the world, it had previously been introduced in the face of the rapidly changing global, economic, technological, and social environment to remain competitive and efficient globally (Lee and Corbett, 2006). However, the perceived outcomes of downsizing were not reaped by the majority of these organizations that faced the dysfunctional consequence of downsizing as survivor syndrome. Survivor syndrome refers to the psycho-social problems such as increased anxiety due to uncertainty, feeling of loss, and risk aversion in employees who have survived cut-offs; employees exhibit survivor syndrome as a decrease in performance, increase in turnover, and absenteeism. Previous studies have claimed that the main reason for this behavior is a decline in affective commitment. Affective commitment is the emotional attachment of an employee with the organization and contributes significantly to maintaining an employee’s performance (Brockner et al., 1993; Cascio, 1993; Hui and Lee, 2000; Lee and Corbett, 2006). On the other hand, after the downsizing phase, the organizations heavily rely on survivors’ performance due to added tasks and responsibilities (Whitener and Walz, 1993; Hackett et al., 1994; Lee and Corbett, 2006). Hence, maintaining the affective commitment of survivors (stocktickerACS) is critical for the organizations to achieve the underlying downsizing goals, including enhancing organizational efficiency (Lee and Corbett, 2006).

Previous research has offered several studies explaining the mechanism through which layoffs affect ACS (Lee and Corbett, 2006; Travaglione and Cross, 2006). However, with the emergence of the new situation of COVID-19, when many organizations used downsizing as a first resort to cover the losses accrued due to a decreased demand worldwide, the causal mechanism of downsizing on survivors’ affective commitment is still unknown (Blustein and Guarino, 2020). Realizing the intensity of need, the objective of this study is to examine the causal mechanism through which downsizing due to COVID-19 (COV-DS) affects ACS.

In the context of the effect of layoffs on survivors, Appelbaum et al. (1997) claimed that the process of downsizing creates the sense of extreme job uncertainty stress in survivors. Job uncertainty stress implies the worry of the survivors about how long they would be able to keep their jobs (Appelbaum et al., 1997). This fear becomes more intense in the challenging and highly uncertain situation of COVID-19, as employees have a realization that they will not be able to find comparable jobs anywhere else due to a shortage of demand. Hence, job uncertainty stress in survivors as a result of COVID-19 (JUSS) is more severe and consequently affects the survivors’ attitudes, such as ACS (Appelbaum et al., 1997; Tu et al., 2021). Hence, the JUSS is expected to play a mediating role betwemetricconverterProductID19 inen COV-DS and declining ACS.

Moreover, Van Dick et al. (2016) asserted that another basic sense that survivors lose at the time of downsizing is organizational identity. Organizational identification is a concept of an employee’s self-referential aspect of identity. Organizational identity becomes a social identification and a source of self-esteem for an employee (Ahuja et al., 2021). Hence, losing organizational identity is considered as losing one’s social identity, consequently lowering an employee’s affective commitment to the organization (Van Dick et al., 2016). Previous research has established the mediating role of declining organizational identification in survivors (OIS), in the relationship of downsizing and affective commitment (Van Dick et al., 2016). Following the same line, this study proposes that as in the other circumstances, also in the times of COVID-19, OIS plays mediating role between COV-DS and ACS.

Management scholars have also averred transformational leaders (TL) that play a significant role in this situation (Bass, 1985; Gentry, 2005; Iftikhar et al., 2021). The TL can positively influence followers’ attitudes by practicing the inherent qualities of a transformational leader, which includes clear communication with subordinates to remove the feeling of uncertainty, the inclusion of subordinates in decision making to maintain their identification with the organization, focusing on the intrinsic motivation, and self-actualization of followers (Bass, 1999; Gentry, 2005). Transformational leaders take care of all the individuals within the organization in the change process, such as downsizing, and ensure that the changes are accepted amicably by all the surviving members in the organization without losing their sense of identification (Herzin and Jimmieson, 2006). Survivors perceive the leader’s support as valuable support. Hence, it mitigates the effect of COV-DS on JUSS and OIS to a greater extent (Kark et al., 2018). Therefore, TL is also proposed to play a moderating role in the challenging situation of COVID-19.

This study is supported by the conservation of resource theory (COR), which states that if employees encounter resource depletion at their workplaces, they cope with it by changing their attitudes and seeking support from any organizational factor (Halbesleben et al., 2014). Based on the theory this study posits that COV-DS, as a resource depletion factor, affects attitudes, such as an increase in JUSS and decrease in OIS, which consequently leads to reduced ACS of survivors with the organization, and they consequently seek help from their respective leaders. This study, therefore, extends the principle of COR to examine the following: (a) the mediating role of JUSS and OIS between COV-DS and ACS, and (b) the moderating role of TL on the relationship of COV-DS and the two moderators (JUSS and OIS).

The present study significantly contributes to the existing literature. First, this study addresses the need for immediate action to minimize the potentially disastrous impacts of COVID-19 in organizations (Van Bavel et al., 2020). This study empirically investigates how inevitable COV-layoffs affect survivors’ AC *via* JUSS, OI, and TL. This manuscript is one of the earliest attempts to explore how COV-layoff lowers survivors’ AC at the workplace in the COVID-19’s context in the private sector, that has been disproportionately affected by COVID-19, and a number of them have chosen a downsizing approach (Tu et al., 2021). This study adds to the literature by investigating the mediating role of OI and JUSS and moderating role of TL attenuating the adverse effect of COV-layoff.

Second, this study deconstructs the process behind COV-layoff and AC using COR theory. Existing research has relied mostly on transactional frameworks to explain layoffs survivors’ reactions (Abuelnasr, 2020; Alaparthi and Thakare, 2020; Bilotta et al., 2020; Eichenauer et al., 2021). Nonetheless, as resources are critical for layoff survivors, COR theory provides a vital theoretical perspective to comprehend the COV-layoff effects on employees’ attitudinal outcomes (Tu et al., 2021).

Pakistan is a developing country, already fighting economic and employability issues. While the emergence of COVID-19 has worsened the situation. As of now, the government cannot provide any kind of financial aid to the unemployed (Akhtar et al., 2020; PIDE, 2020). This makes the problem of downsizing critical for the country, which is only second to the health crises in the times of COVID-19. In the current situation, ignoring this issue is as dangerous as dismissing the coronavirus. Pakistan Institute of Development Economics (PIDE) previously estimated job losses from COVID-19 at up to 18.5 million (PIDE, 2020). In this situation, the only option for the organizations to survive is to keep up the commitment of surviving employees by removing the insecurities that hinder their performance. By observing the critical situation, this study is based on the data collected from Pakistani employees who have survived the layoffs.

The next section discusses the COR theory, the theoretical framework, and, subsequently, research hypotheses are developed.

## Theory and Hypotheses Development

### Conservation of Resource Theory

The conservation of resource theory (COR) (Hobfoll, 1989) serves as the theoretical underpinning for this investigation as it provides a helpful framework for understanding human well-being and stress (Westman et al., 2004). The COR is based on two principles: first is that employees protect the resources (any valuable object, condition, or state) from being lost because losing a resource or having a threat of losing it will drive individuals into certain levels of stress. These resources may include physical resources (e.g., homes, clothing, foods, and transportation), psychological and social resources (e.g., psychological resilience, social networks, job security, social support, self-efficacy, social identification, or self-esteem), personal disposition, and cultural routinization (e.g., job experience, gender, and marital status). The principle also establishes that losing a resource has a more damaging effect than gaining it because it may negatively influence one’s in-role and extra-role performance (Hobfoll, 1989; Halbesleben et al., 2014). Moreover, the initial resource loss will further lead to resource loss in the future (Hobfoll, 1989). The second principle states that employees, on losing a particular resource, will tend to adopt coping mechanisms by investing in other resources to protect, recover, and gain them (Hobfoll, 1989). Grounded in COR, the current study posits that COV-DS, as an initial resource loss for survivors, in the form of losing social networks, which instigates stress as JUSS and further resource loss as OIS, consequently leads to a decline in extra-role behaviors that are indicated by ACS (Lin et al., 2021). The COR also asserts that employees suffering from resource depletion seek coping mechanisms by utilizing other resources, such as a leader’s support. In this regard, management scholars have averred that transformational leaders can act as a coping mechanism (Bass, 1999; Gentry, 2005).

### Downsizing Due to COVID-19 and Affective Commitment of Survivors

Classical studies have introduced the dysfunctional consequences of downsizing as survival syndrome, which indicates that the major factor that contributes to the failure to achieve strategic objectives of downsizing is ignoring the emotional state of survivors (Schweiger et al., 1987; Isabella, 1989; Appelbaum et al., 1997). These studies have strongly asserted that survivors in the organization suffer adverse emotional effects after the occurrence of downsizing, such as damaged affective commitment that the survivors have to the organization (Schweiger et al., 1987; Isabella, 1989; Appelbaum et al., 1997). Explaining the phenomenon, Schweiger et al. (1987) stated that survivors perceive that organization has not treated the committed employees with respect and dignity to which they are entitled. Consequently, they lower their emotional attachment and affective commitment to the organization.

Prior research hasmetricconverterProductID19 in also established that downsizing leaves a stressful impact on survivors (Mishra and Spreitzer, 1998; Tang and Ibrahim, 1998; Boyd et al., 2014). As they perceive downsizing as an irrevocable loss of close coworkers, cause of increased work-load, and job uncertainty (Tang and Ibrahim, 1998; Hui and Lee, 2000; Quinlan and Bohle, 2009; Spagnoli and Balducci, 2017). These stressors become a cause of the declining ACS to their respective organizations (Spreitzer and Mishra, 2002; Lee and Corbett, 2006; Jiang et al., 2020). Travaglione and Cross (2006) asserted that affective commitment is known to be the strongest and the most significant among the three dimensions of commitment including affective (emotional attachment), continuance (attachment due to monetary benefits), and normative (attachment due to moral obligations) (Travaglione and Cross, 2006). Also, contrary to others, due to its sensitive nature, Affective commitment tends to be afflicted the most due to downsizing (Travaglione and Cross, 2006). The phenomenon was also supported by Cappelli (2000), he stated that in the situation like downsizing, the commitment is not totally lost, in fact, only ACS get negatively influenced, which is the commitment based on emotions and loyalty and it is replaced by the commitment built on self-interest and economic benefits.

Recent studies have also claimed that the new scenario developed by COVID-19 become the cause of declining ACS, as the organizations need to change the structures and policies accormetricconverterProductID19. Indingly. These unexpected changes compounded by the unprecedented situation of COVID-19 become the cause of psychological problems and loss of emotional commitment in employees (Wiesenfeld et al., 2001; Mihalache and Mihalache, 2021). Scholars have established that environmental disruptions, like the recent pandemic of COVID-19, tend to erode affective commitment, and, hence, the overall work-related well-being of employees gets affected. Therefore, maintaining affective commitment is crucial for the survival of organizations (Wiesenfeld et al., 2001; Mihalache and Mihalache, 2021). Following the same lines, based on COR, which states that losing a resource leads to an employee’s decrease in both in-role and extra-role behaviors (Halbesleben et al., 2014), the current study proposes that the layoffs occurred due to the specific situation of COVID have an adverse effect on ACS of survivors. Hence, it is expected that:

*Hypothesis 1*: The COVID-19-downsizing has a negative association with the ACS.

### The Mediating Role of Job Uncertainty Stress in Survivors

Prior studies based on COR have established that in an organization, downsizing becomes the major source of mental stress in survivors (Morelli and Cunningham, 2012). As for the survivors, downsizing results in depletion of two types of resources: first, the social support of the friends/coworkers, and second, the job security (Grunberg et al., 2001; Brockner et al., 2004; Van Dierendonck and Jacobs, 2012). The COR further implies that in response to the stress that occurred due to downsizing, survivors tend to adopt a coping mechanism to prevent the remaining resources (Morelli and Cunningham, 2012; Halbesleben et al., 2014). These coping mechanisms may include a change in attitude or in-role/out-role behaviors (McCarthy et al., 2019; Lin et al., 2021).

In compliance with previous research, recent studies in the context of COVID-19 have also established that COV-DS triggers job uncertainty stress in survivors due to its unprecedented and unpredictable nature, which further leads to change in survivors’ attitudes (Mujtaba and Senathip, 2020; Tu et al., 2021). Following the same line, this study proposes that when observing COV-DS in their organizations, survivors manifest psychophysiological reactions against loss of valued resources as JUSS, which becomes the source of declining AC in them. Hence, it is proposed that:

*Hypothesis 2*: The JUSS mediates the negative relationship of downsizing due to COVID-19 and ACS.

### The Mediating Role of Organizational Identification of Survivors

Management scholars have asserted that other than individual identity, people think, act, and take pride as a member of a social group. Likewise, employees from an organization gain this social identity from the organizational membership that further enhances their pride and self-esteem. Hence, organizational members strongly value the recognition they get as part of an organization. It gives them the motivation to stay and remain loyal to it (Tajfel and Turner, 1979; Ashforth and Mael, 1989). Conversely, if employees think that organization is not reciprocating and has adopted the downsizing strategy, they lose the connection with the organization in the form of lowering identification with it, which results in declining in-role and extra-role performances (Riketta, 2005). In this regard, scholars have averred that by opting for the downsizing strategy, employers may create disappointment among employees; the employees who have survived the cut-offs might get the impression that the organization is unempathetic toward its employees. Working for such an organization may, thus, generate a negative social identity in them. Hence, they reduce their identity with the organization, which further leads to reduced ACS (Allen et al., 2001; Travaglione and Cross, 2006; Van Dick et al., 2016). In the same context, Van Dick et al. (2016) established that downsizing hits survivors’ identification with the organization and further to ACS, as both attitudes are interconnected and generated from the same basis that is an emotional attachment with the organization.

Moreover, the scholars identified the gap in studies on organizational identification and emphasized the need to study it more in the context of downsizing (Van Dick et al., 2016; Campos-García et al., 2020). The fragility of psychological health has led researchers to investigate the notion of OIS as an antecedent of affective organizational commitment (Bednar et al., 2020). According to Tufan and Wendt (2020), the OIS of employees is a critical predictor of variance in employees’ attitudes. To cope with high-pressure decisions, such as COV-DS, organizations must have survivors with strong OIS to confront the hyper turbulent situation. To the best of our knowledge, organizational identification has not been studied in the context of COVID-19 yet. Realizing the gap, this study claims that COV-DS, just as downsizing due to other factors, becomes the source of decreased OIS in survivors. Hence, consequently, they change their attitudes in the form of lowering ACS with the organization (Hogg and Terry, 2000).

The COR provides strong support for the mediating relationship of OIS between COV-DS and ACS. According to this theory, employees feel less absorbed into the organization when they feel that they are losing a resource due to the organization’s strategy. This feeling engulfs their positive storage of energy and they are left with little positive emotions for the organization (OIS), which can have an impact on their affective commitment to the organization (Van Knippenberg and Sleebos, 2006). Particularly, laying off the employees propagates the less empathetic behavior of employers; consequently, the lowered OIS is expected to influence the ACS of survivors. Hence it is expected that:

*Hypothesis 3*: The OIS mediates the negative relationship between downsizing due to COVID-19 and ACS.

### The Moderating Role of Transformational Leadership

The COR argues that gaining resources in a highly stressful situation becomes more salient to cope with it (Gentry, 2005). Based on COR, Hobfoll et al. (2018) established that COV-DS has emerged as a strong stressor that causes the sense of lost resources, not only for laid-off employees but for survivors too. Consequently, survivors look for factors like organizational support to balance the resources (Carlson and Perrewé, 1999). A leader is one of the significant organizational factors, that is considered as representative of an organization by subordinates, and, hence, survivors seek psychological and physical support from the leader to minimize the survivor’s syndrome, such as JUSS and loss of OIS (Sluss et al., 2008; Anser et al., 2021).

The scholars established that TL can be strong influencers to maintain subordinates’ positive attitudes toward the organization (Avolio et al., 1991; Bass and Avolio, 1993; Sluss et al., 2008). In the same context, Jain and Duggal (2018) claimed that subordinates working under transformational leaders have increased belief that they play a pivotal role in the company and, hence, they feel more motivated, secure, and identify more with the company; consequently, build a stronger commitment toward it (Jain and Duggal, 2018). Hogg (2001) further stated that transformational leaders are proactive, inspiring, and change-oriented. They are expected to involve, inspire, and motivate subordinates to maintain their positive attitudes toward work during the change process. In this regard, Matsunaga (2021) established the indirect effect of transformational leadership during times of uncertainty on subordinates’ attitudes and performance. The scope of this study is specific to two of the significant behaviors, OI and JUSS, which are expected to be affected in the COVID-19 scenario. Both the attitudes will be discussed in the context of transformational leadership in the following paragraphs.

In the context of JUSS, Tu et al. (2021) argued that the COV-DS experience is emotionally wrenching for both laid-off employees and survivors, though the survivors have not faced cut-offs it leaves job uncertainty stress in them. Tu et al. (2021) further stated that the feeling of uncertainty stress in survivors is inevitable because of the way victims were treated and their job security is jeopardized. It generates the feeling in them that they could be the next victim of downsizing (Kim, 2003). In such a situation, classical literature claimed that keeping the survivor’s engagement highly depends on the fact that how the higher management handles the situation (Appelbaum et al., 1997). In this regard, Appelbaum et al. (1997) suggested the significant role of leaders, who should be able to maintain survivors’ commitment to the organization, communicate with them the causes of downsizing well, keep the survivors informed about the changes at each step, and involve them in decision making. A transformational leader can serve all the purposes by applying the inherent qualities that make him/her transformational, (a) giving individualized consideration, so survivors can feel an important part of the organization, (b) involving the survivors in the change process through intellectual stimulation, (c) motivating subordinates to perform well through inspiration, and (d) influencing them positively through establishing a strong relationship with them (Walumbwa et al., 2008). Hence, we expect that transformational leadership moderates the relationship between the COV-DS process and JUSS.

Taking OIS into consideration, Ashforth and Mael (1989) stated that literature provides numerous reasons to expect that TL will enhance the OIS of employees during different times in the life cycle of an organization. Scholars established that transformational leaders have strong influencing power that affects the subordinates’ concept of self-identity; subordinates associate their identity with the identity of the organization, and, hence, take pride in the organization’s success (Lord et al., 1999; Bass, 2000; Hogg, 2001). Likewise, Dvir et al. (2002) established that developing transformational leadership in supervisors directly influences subordinates’ identification with the organization and consequently, indirectly affects subordinates’ attitudes and performance. Ribeiro et al. (2018) claimed that the organizational changes that are affecting employees adversely decrease their pro-organizational attitudes and a poor leadership adds to the situation negatively. Conversely, transformational leadership may compensate for the negative impacts of unfavorable circumstances through executing the change amicably (Den Hartog et al., 1997; Bass, 1999). Furthermore, the employees who get organizational support, such as the leader’s support, felt more identified with it and have a greater degree of devotion to perform for their companies (Liu et al., 2019). Hence, we expect that TL moderates the relationship between the COV-DS and OIS, in a way that a higher TL will buffer the negative effect of COV-DS on OIS.

*Hypothesis 4*: The TL moderates the relationship between downsizing due to COVID-19 and JUSS, such that transformational leadership will weaken the positive effect of downsizing due to COVID-19 on JUSS.
*Hypothesis 5*: The TL moderates the relationship between downsizing due to COVID-19 and survivors’ organizational identification such that transformational leadership will minimize the negative effect of downsizing due to COVID-19 on OIS.

## Methodology

### Samples and Data Collection

We collected the data from private sector organizations of Pakistan. Initially, we obtained the e-mail addresses of working professionals of different private sector organizations. The link to the survey questionnaire was sent to 500 people *via* e-mail. The survey questionnaire included 41 items for measuring the study variables, and 5 questions on demographic variables were also included. Two hundred seventy-four usable questionnaires were obtained, which makes the response rate at 54%.

### Measures

All scales were rated from (1 = strongly disagree to 5 = strongly agree) except for the downsizing scale, which was rated as (1 = Agree, 2 = disagree). The downsizing scale used was developed by Moore et al. (2004). The sample items include: (1) My close friends in the company were laid off due to COVID-19; (2) Some of my coworkers were laid off due to COVID-19. The organizational identification scale of Park and Back (2020) was used. The sample items include: (1) Being a part of my current organization is important to me; (2) I feel a strong sense of belonging to my current organization. The job uncertainty scale of Vander Elst et al. (2014) was used. The sample items include: (1) Due to COVID-19, there are chances that I will soon lose my job; (2) Due to COVID-19, I feel insecure about the future of my job. The affective commitment scale of Allen and Meyer (1990) was employed having the following sample items: (1) I enjoy discussing about my organization with people outside it; (2) I do not feel like part of the family at my organization. The transformational leadership scale of Carless et al. (2000) was used. The sample items consist of: (1) My boss communicates a clear and positive vision of the future during the times of COVID-19; (2) My boss treats staff as individuals who support and encourage their development during the times of COVID-19. We used age, gender, education, and experience as control variables as they contaminate the relationship between the dependent and independent variables (Shaw et al., 2009).

### Analysis and Results

The normality assumption of the data set was checked by using Shapiro–Wilk’s *W* test (recommended: *p* > 0.05), as well as histograms, box plots, and normal *Q*–*Q* plots, were checked which showed that data was non-normally distributed. Thus, we used PLS-SEM by WarpPLS 7 (http://warppls.blogspot.com/, Kock, 2018). For controlling common method bias (CMB), we employed full collinearity tests at the analysis stage (Kock, 2015). As a result, we found values of the variance inflation factor (VIF) for all the acceptable study variables (VIF ≤ 5), i.e., 1.2–1.8 (Kock, 2018). Finally, the average block variance inflation factor (AVIF = 1.26) and average full collinearity variance inflation factor (AFVIF = 1.40) were found to be ideal (≤3.3), respectively. These values indicate no serious effect of CMB on our findings. The goodness of fit indices was checked and all indices (Classic fit indices and Additional fit indices) indicate that data is fit for measurement. While employing reflective measurement, we analyzed Tenenhaus goodness of fit (GoF = AVE × *R*^2^). Results revealed GoF = 0.52, indicating a good fit of our model, as GoF ≥ 0.10, 0.25, and 0.36 is considered small, medium, and large, respectively. These results are substantiated by some additional indices, i.e., standardized root mean square residual (SRMR = 0.08; Benchmark is ≤1), Standardized mean absolute residual (SMAR = 0.09; ≤1), and *R*-squared contribution ratio (RSCR = 1; ≥0.90). Reliability estimates of Cronbach α values (0.75–0.92) and composite reliability coefficients (ρc) (0.84–0.94) are satisfactory, at ≥0.70 (see Table 1). For establishing the convergent validity of constructs, all factor loadings at the indicator level were checked and found to be satisfied (>0.7, *p*s < 0.001). Secondly, the values of average variance extracted (AVE) for all variables satisfied the traditional criterion, AVE ≥ 0.50 (0.51–0.80) (see Table 1).

**TABLE 1:** Latent variable estimates.

Variables	*R* ^2^	Adjusted *R*^2^	*ρ_*c*_*	α	AVE	VIF	*Q* ^2^
COVID-downsizing	—-	—-	0.88	0.81	0.65	1.83	—-
Job uncertainty stress	0.44	0.44	0.94	0.91	0.80	1.70	0.43
Organizational identification	0.05	0.05	0.94	0.93	0.58	1.21	0.06
Affective commitment	0.48	0.47	0.84	0.78	0.51	1.36	0.48

*ρ_c_, Composite Reliability; α, Cronbach’s alpha; AVE, Average Variances Extracted; VIF, Variance Inflation Factor (Full collinearity); Q^2^, Predictive Relevance.*

For establishing divergent validity, first, we found all values of √AVE greater than correlation coefficients in their corresponding rows and columns (see Table 2). The second method used is the Heterotrait-Monotrait (HTMT) ratio of correlations, which should be less than 0.85 (Henseler et al., 2015), and we got satisfactory values for all constructs. Lastly, for measuring predictive validity, all coefficients of predictive relevance were found to be nonzero, *Q2*≠ 0 (see Table 1). Therefore, the internal validity of all constructs is established.

**TABLE 2:** Mean, SD, inter-correlations, and Average Variance Extracted (AVE).

Variables	*M*	*SD*	1	2	3	4	5
COVID-downsizing	1.45	0.39	**0.81**				
Job uncertainty stress	2.94	0.88	0.55**	**0.89**			
Organizational identification	2.09	0.62	−0.10*	–0.14*	**0.76**		
Affective commitment	3.55	0.55	−0.24**	−0.27**	0.23**	**0.71**	
Transformational leadership	3.72	0.71	−0.12*	−0.07	0.20**	0.33**	**0.80**

*p < 0.05, **p < 0.01, *** p < 0.001, $\sqrt{\text{AVE}}$ in bold face are shown on diagonal.

### Hypothesis Testing

Hypothesis 1 pertains to the relationship between COV-DS and ACS. The results in Table 3 indicate that COV-DS has negatively affected ACS (path c) (β = −0.30, *t* = −5.35, *p* < 0.001). Hence, Hypothesis 1 is supported. Hypothesis 2 proposes the mediation effect of JUSS on COV-DS and ACS relationship. The results in Table 3 indicate that the relationship between COV-DS and JUSS (paths *a*_1_) is positive and significant (β = 0.64, *t* = 11.84, *p* < 0.001). Moreover, the relationship between JUSS and ACS (path *b*_1_) is negative and significant (β = −0.28, *t* = −5.02, *p* < 0.001). Furthermore, the relationship between COV-DS and ACS (path *c’*) is reduced in magnitude after controlling for JUSS (β = −0.13, *t* = −2.28, *p* < 0.001). The confidence intervals also show the significantly indirect effect indicating mediation process [*B* = −0.17, *SE* = 0.07, CI_95%_ (−0.327; −0.03)]. Therefore, *H2* is supported.

**TABLE 3:** Mediation and moderation results.

	COV-DS→JUSS→ACS COV-DS→OIS→ACS		
	*B(SE)*	*t*	CI_95%_
**Direct effects**			
Path *a*_1_ (Direct effect of COV-DS on JUSS)	0.64(0.05)	11.84***	
Path *a*_2_ (Direct effect of COV-DS on OIS)	−0.15(0.05)	−2.53*	
Path *b*_1_ (Direct effect of JUSS on ACS)	−0.28(0.05)	−5.02***	
Path *b*_2_ (Direct effect of OIS on ACS)	0.35(0.05)	6.20***	
H1: Path c (Total effect of COV-DS on ACS)	−0.30(0.05)	−5.35***	
Path c’ (Total effect of COV-DS on ACS, controlling for JUSS)	−0.13(0.05)	−2.28***	
Path c’ (Total effect of COV-DS on ACS, controlling for OIS)	−0.32(0.05)	−5.58***	
**Indirect effects**			
H2:M_1 COV–DS→JUSS→ACS_	−0.17(0.07)		[−0.32; −0.03]
H3:M_2 COV–DS→OIS→ACS_	−0.01(0.01)		[−0.05; 0.011]
**Moderated mediation**			
H4: TL*COV-DS (COV-DS→JUSS→ACS)	−0.21(0.05)	−3.65***	[−0.32; −0.09]
H5: TL*COV-DS (COV-DS→OIS→ACS)	−0.18(0.05)	−3.20***	[−0.30; −0.07]

*p < 0.05, **p < 0.01, ***p < 0.001, ns, not significant, N = 274, CI_95%_, Confidence Interval at 95%; COV-DS, Downsizing due to COVID-19; JUSS, Job Uncertainty Stress in Survivors; OIS, Organizational Identification of Survivors; ACS, Affective Commitment of Survivors; TL, Transformational Leadership.

**FIGURE 1:**
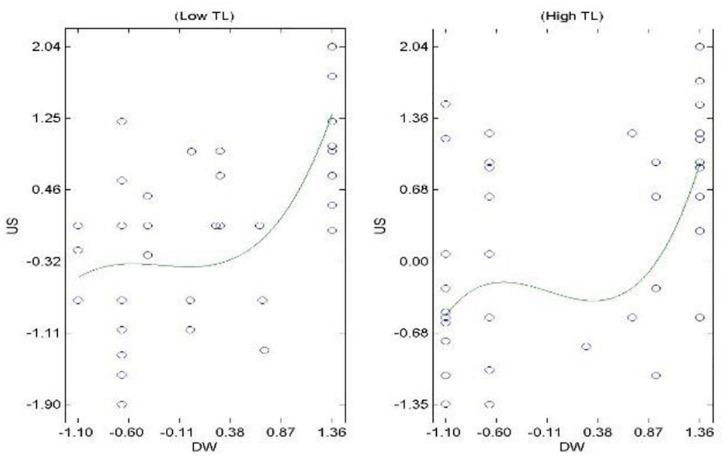
Moderation of TL on the relationship of COV-DS and JUSS.

**FIGURE 2:**
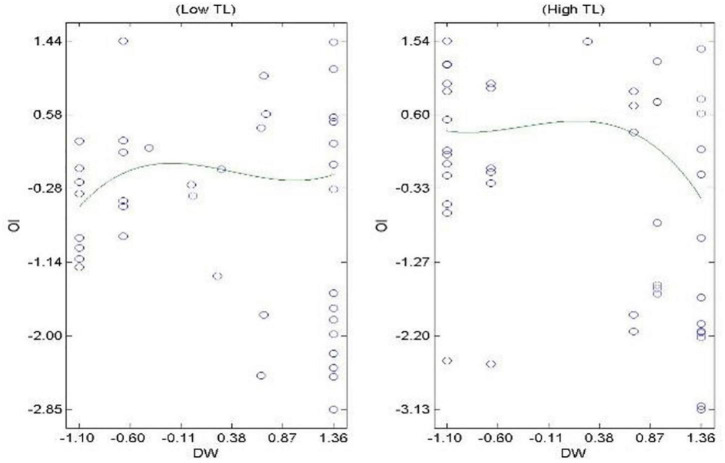
Moderation of TL on the relationship of COV-DS and OIS.

**FIGURE 3:**
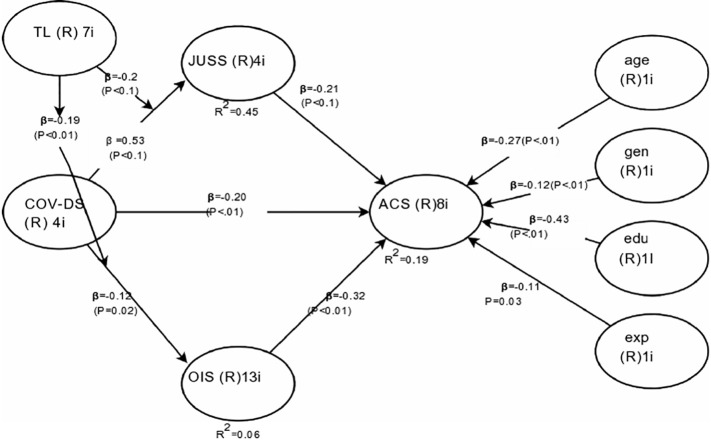
Theoretical framework of the study.

Hypothesis 3 proposes the mediation effect of OIS on the COV-DS and ACS relationship. The results in Table 3 indicate that the relationship between COV-DS and OIS (paths *a*_2_) is negative and significant (β = −0.15, *t* = −2.53, *p* < 0.05). The relationship between OIS and ACS (path *b*_2_) is positive and significant (β = 0.35, *t* = 6.20, *p* < 0.001). The relationship between COV-DS and ACS (path *c’*) is not reduced in magnitude after controlling for OIS (β = −0.32, *t* = −5.58, *p* < 0.001). This result does not provide initial support for mediation. Furthermore, the confidence intervals show the insignificantly indirect effect indicating no mediation process (*B* = −0.01, *SE* = 0.01, CI_95%_ [−0.05; 0.01]). Therefore, *H3* is not supported. The H4 pertains to the moderation effect of TL on the relationship of COV-DS on ACS *via* the mediation of JUSS. Results in Table 3 indicate the acceptance of Hypothesis 4. The H5 proposes the moderating effect of TL on the relationship of COV-DS and ACS *via* the mediation of OIS. Results in Table 3 pertain to the acceptance of H5. Figure 1 demonstrates the graphical representation of the moderation effect of TL on the relationship of COV-DS and JUSS that further leads to AC. The graph shows that when TL is low, the JUSS reaches the level of 1.25; when the TL is high, the JUSS level just reaches 0.7. Hence, we can conclude that TL plays a moderating role to bring down the level of JUSS as a result of COV-DS. Figure 2 shows that at a lower level of TL the effect of COV-DS on OIS would be −0.3 and quite stable. On the other hand, when the TL is high, the level of OIS may reach the level of 0.58. Hence, we can say that TL mitigates the effect of COV-DS on OIS until it reaches a certain limit. Figure 3 displays the theoretical framework of the whole model. It depicts the significant effect of both moderated mediation (a. Moderated mediation of TL and COV-DS on JUSS that leads to AC, and b. Moderated mediation of TL and COV-DS on OIS that leads to AC). The figure confirms the results that TL mitigates the effect of COV-DS on JUSS and OIS that further leads to enhanced ACS.

## Discussion

The COVID-19 has a negative effect on every industry around the globe, as it leads to a decrease in demand and, consequently, organizations are forced to downsize (Blustein and Guarino, 2020). This situation has imposed an immense adverse effect on both laid-off and surviving employees (Blustein and Guarino, 2020; Dirani et al., 2020; Nicola et al., 2020). Consistent with prior literature on downsizing, the COV-DS literature also stated that after downsizing, the prime concern of organizations are survivors because the performance of the organizations depends on the attitudes and behaviors of survivors, who are in the state of uncertainty and trying to cope with the changes (Kim, 2003). Grounded in COR, this study unearths the causal mechanism of COV-layoffs on survivors’ most critical attitude: the affective commitment, as it becomes the basis for several other positive attitudes and behaviors (Lee and Corbett, 2006).

This study tested four effects: (a) the direct effect of COV-DS on ACS, (b) the mediating role of JUSS between COV-DS and ACS, (c) the mediating role of OIS between COV-DS and ACS, and (d) The moderating role of TL between COV-DS and both the mediators (JUSS and OIS).

The significant and negative relation of COV-DS and ACS (Table 3), established by hypothesis 1, is consistent with the previous literature. Affective commitment primarily entails internal motivational factors, including attachment and involvement of an employee with the organization. Other commitment dimensions are as follows: continuance and normative are based on economic and ethical values, respectively (Mayer et al., 1993; Metcalfe and Dick, 2000). The dimensions based on external motivational factors may not change for survivors, during downsizing (Metcalfe and Dick, 2000). The external factors, such as monetary benefits, supervisor, and organizational support, remain the same. On the other hand, ACS disrupts by the psychological turmoil involved during downsizing by losing social capital in form of friends and colleagues, facing uncertainty stress, observing the inhuman treatment of the organization with laid-off employees, and handling the increased burden of work (Kim, 2003). Hartmann and Bambacas (2000) claimed that affective commitment among survivors is the only dimension of commitment that gets affected negatively as a result of downsizing the other two dimensions of commitment remain the same or even get stronger.

This study also proposed the mediating effect of JUSS and OIS between COV-DS and ACS (Hypotheses 2 and 3, respectively). The results (Table 3) indicate that Only JUSS mediates the relationship of COV-DS and ACS, such that, COV-DS increases the JUSS in survivors and as a reaction, they decrease their ACS. On the other hand, OIS does not play mediating role between COV-DS and AC. The strong mediating effect of the JUSS can be explained by the fact established in prior literature that the bizarre circumstances of COV-DS appear to be a strong element in triggering uncertainty stress and bringing down the positive behaviors among surviving employees (Kang et al., 2021). The overall life and future uncertainty, due to the unprecedented situation of COVID-19, adds up to the job uncertainty factor, as each day brings new and bigger challenges for organizations and individuals working in them (Bufquin et al., 2021). On the other hand, the behavior of OI can be explained by the fact that layoffs like in the situation of COVID-19, which have a strong rationale behind it, have a smaller effect on organizational identification of survivors, as compared to the layoffs being done solely for-profit maximization purpose. The survivors perceive downsizing strategies in such situations as justifiable and adapt to them amicably without losing much of their identification with the organization (Van Dick et al., 2016).

This study also proves the moderating role of transformational leadership on the relationship of COV-DS and both the attitudes, (a) JUSS and (b) OIS (Hypotheses 4 and 5). This behavior has its roots in previous studies on leadership, which have established that effective leadership contributes to stabilizing the employees’ identification and decreases the feeling of uncertainty during times of downsizing by exercising the practices inherent in transformational leadership. These practices include supporting the subordinates in the downsizing process, including them in the decision-making process by giving them clarity about the goals of an organization, motivating them to accept the change, and developing the sense of their worth for the organization (Ullrich et al., 2005; Sluss et al., 2008; Van Dick et al., 2016).

The moderating effect of TL in the relationship of COV-DS and the JUSS has its roots in COR, as the theory establishes that resources play an essential role in understanding employee stress in the layoff situation (Bono and Anderson, 2005). The findings of this study are consistent with the COR proposition regarding coping with conditions characterized by a lack of resources (Halbesleben et al., 2014). Exposure to the threatening situation (COV-DS) by survivors worsens their psychological resources (causing JUSS), which leads to the adoption of defensive attempts to conserve resources; for example withdrawing the emotional attachment (Avolio and Bass, 1995). Individuals in the uncertainty stress reported that receiving social support helps to decrease their stress during the downsizing phase. In this regard, TL is proven to be a fundamental component that can uplift the positive attitudes among the survivors (Avolio and Bass, 1995). Transformational leadership replenishes the lost resources and energy within the survivors and minimizes the effect of stress among employees. This study is consistent with prior studies that highlighted the significant role of TL as a social resource in alleviating the harmful effects of COV-layoff on ACS through affecting the JUSS (Walsh et al., 2014).

Moreover, the management literature establishes that one factor that gets affected after downsizing is the organizational identification of surviving employees (Riketta, 2005). Though this study proves that OIS is not moderating between COV-DS and ACS, as the survivors thought their organizations’ attribution in COV-DS is not much, hence, they do not lose much identification with the organization (Van Dick et al., 2016). However, transformational leaders, realizing the importance of OIS in building ACS, can utilize OIS to enhance ACS. This study establishes that when TL comes into play as a moderator between COV-DS and OIS, the mediation effect of OIS becomes active. This can be justified through the literature on social identity, which suggests that the qualities of transformational leaders make them capable of generating a stronger organizational identity among subordinates, which gives them a drive toward a common organizational goal (Ashforth and Mael, 1989; Hogg, 2001; Kark and Shamir, 2002; Van Knippenberg et al., 2004). Hence, transformational leaders play a very significant role in utilizing OIS to affect ACS positively. Moreover, grounded in the theoretical argument built by prior literature that the organizational identification satisfies employees’ esteem and predictability needs, a transformational leader moderates the effect of COV-DSS on OIS by realizing these needs throughout the downsizing process. He/she develops a strategy to minimize the damages. This strategy includes building a strong relationship with survivors, communicating the need for downsizing, motivating, and influencing them to accept the changes (Walumbwa et al., 2008).

Furthermore, this study was conducted under the specific boundary conditions of Pakistan. The results established that COV-DS has a strong effect on JUSS; this behavior can be explained by the circumstances prevailing in the country, as Pakistan is already facing weak financial conditions, unemployment, high inflation, and low-income per capita issues (PIDE, 2020). In this situation, COVID-19 has worsened the situation because the employees who have even survived cut-off still have fears of uncertainty. They realized the fact that if the situation of COVID-19 prolongs, they could be the next ones to get laid off (Blustein and Guarino, 2020). The fear becomes even more severe when alternate jobs or government support is not available, such as in a developing country like Pakistan.

### Theoretical Implications

This study has significant academic implications. First, this study will add significantly to the literature on the harmful effects of COVID-19 on the organizations’ performance. The COVID-19 is an unprecedented and unknown phenomenon that has forced many organizations to downsize to survive (Javed et al., 2020). To combat the situation, it is, therefore, a need to know whether and how COV-layoff influences employee outcomes. Tu et al. (2021) claimed that there are numerous studies in the context of COVID-19 that have found the impact of downsizing on laid-off employees. Tu et al. (2021) were the first ones to study the effect of COVID-19-induced layoffs on survivors. However, their research was restricted to one attitude (stress) and industry (hospitality industry) only. They emphasized the pressing need of exploring more about the COVID-19-induced effects on organizations and people working in them. Following the same line, our study extended the literature by testing an underlying mechanism through which COV-DS affect ACS.

Second, a previous research based on COR has shown the value of TL in dealing with traumatic life situations (Middleton et al., 2015). However, to the best of our knowledge, we are the first ones to test the effectiveness of transformational leadership in the specific COVID-19 scenario. The findings supported the proposition that substantial levels of social support from TL may assist survivors to get through this challenging time by minimizing COVID-19-induced stress and preserving organizational identification.

Third, the organizational studies that have explored the effects of COVID-19 claimed that it negatively affects the psychological health, attitudes, and behaviors of employees (Aladejebi, 2020; Eichenauer et al., 2021; Tu et al., 2021). This study that was added in literature by identifying that the effect of COVID-19 on different variables may vary in magnitude. For example, the COV-DS has a stronger effect on JUSS compared to OIS (Table 3) and, hence, should be dealt with accordingly.

Fourth, this study discovered a stress process in which COV-DS is positively linked with JUSS. Prior research studies have mostly looked at the consequences of layoffs on employees’ behavioral outcomes from a justice standpoint (Saad and Elshaer, 2017; López Bohle et al., 2021). The justice perspective cannot be rejected in circumstances where the downsizing process is well-thought and well-implemented. The COVID-19-induced downsizing is not a choice but is inevitable for organizations, where downsizing is not based on justice but a necessity. By taking the specific situation of COVID-19 into account, we contend that resources are critical in comprehending employees’ JUSS in a COV-DS situation. Hence, this study adds to the stress literature by viewing it from the perspective of depletion and gaining of resources, as explained by COR.

Most of the studies on COVID-19 have been conducted in western countries or China, where the economic conditions are comparatively stronger (Blustein and Guarino, 2020; Nicola et al., 2020; Tu et al., 2021). This study will clarify the COVID-19 related attitudes specific to employees working in financially weak countries, such as Pakistan.

### Practical Implications

This study also has several meaningful practical implications. Even though COVID-19-induced layoff is inevitable for many firms around the globe, the employees’ insecurities generated through COV-DS need to be properly addressed (Tu et al., 2021). For example, before taking adverse actions such as downsizing, the management needs to approach employees using effective communication that thoroughly explains the problem and solicits their understanding. We found that TLs can play an effective role in this regard by informing, including, motivating, and influencing the survivors through their inherent qualities that will redress the negative effects of COV-DS up to great extent.

Moreover, this research has been conducted in the private sector, as the private sector has been hit the most by COVID-19. The reason behind this is that the private sector merely strives for profit maximization without any due consideration of the human factor. This stimulates a high level of uncertainty stress in employees working in the private sector (Tu et al., 2021). Likewise, this study also shows high JUSS in response to COV-DS. Thus, the private sector management should make serious efforts to help employees cope with uncertain situations in the times of COVID-19 through training, competency development, and stress management programs. Organizations may impact their survivors’ emotions and behaviors by building confidence, collaboration, and mutual trust. Moreover, the organizations should train the leaders to manage the employees in the uncertain situation of COVID-19 (Brammer and Clark, 2020).

The study will provide a direction to managers in devising policies and planning actions. The managers can target the attitudes that are proven to be most affected due to COV-DS, specifically in developing countries. This is very significant information for managers as their most important task is to maintain the motivation of survivors, which ultimately results in organizational survival (Hui and Lee, 2000; Lee and Corbett, 2006). The study also proved that COV-DS has a greater effect on JUSS as compared to OIS. While devising a strategy for survivors, managers should keep this fact in mind and should stress more on uncertainty stress as compared to OIS. They can do this by arranging expert counseling sessions for survivors, who can directly address their uncertainty issues.

### Limitations and Future Directions

This study is subject to several limitations. First, it is based on a cross-sectional design, as data were collected at one point in time from the same source. The reason for this is that the COVID-19 is a recent phenomenon and longitudinal study on survivors is not yet possible. Even though post-remedial strategies were adopted to check the common method bias (Kock, 2015) and to have a more comprehensive picture, the future studies can use a longitudinal design and multiple sources when collecting data. Second, this study is confined to three attitudes and one leadership style only—JUSS, OIS, ACS, and TL; the future studies can test the attitudes and behaviors other than the aforementioned that can be affected by downsizing as survivors’ syndrome or can affect the survivor’ syndrome; for example, organizational citizenship behaviors, counter-productive work behaviors, perceived organizational support, performance, crises leadership style, and job satisfaction, to understand the complex phenomenon of COVID-19 effect on organizations and people working in them (Hui and Lee, 2000; Quinlan and Bohle, 2009; Boyd et al., 2014; Spagnoli and Balducci, 2017). Third, this study has been conducted in the context of Pakistan, where the reason for high uncertainty stress may be the low employability factor prevailing in the country (PIDE, 2020). The situation might be different in other countries; hence the study can be replicated in other regions of the world. Moreover, an observation from Figure 2 is that the IOS improves due to intervention of TL up to a certain limit, then it starts decreasing. The limit to which the TL improves the OIS in response to COV-DS is out of the scope of this study but future studies can try to identify the limit and causes of this behavior.

Furthermore, the impact of downsizing on the attitudes of employees may be different in different cultures; for example, the reaction may be different in a collectivist country as compared to an individualistic one, due to different mindsets and strength of social support (Matsumoto and Hwang, 2013). The phenomenon of downsizing and its reactions can also be tested by inducing cultural variables.

## Conclusion

Grounded in COR, this study investigated the effect of COV-DS on ACS. This study posits that COV-DS has a negative effect on ACS through the mediating effect of JUSS and IOS, and the moderating effect of TL. The study findings indicate that the direct effect of COV-DS on ACS was negative. The partial mediation effect of JUSS was also proven to be significant, such that COV-DS has a positive effect on JUSS that leads to the negative effect on ACS. On the other hand, the mediating effect of OIS between COV-DS and OIS was not proven as significant. Furthermore, this study established that TL can play an effective buffering role by reducing the positive effect of COV-DS on JUSS and strengthening the negative relationship between COV-DS and OIS. Additionally, by introducing TL as a moderator the mediating effect of both the moderators become significant (JUSS and OIS). The findings also revealed that the effect of COV-DS is more detrimental on JUSS as compared to OIS.

## Data Availability Statement

The raw data supporting the conclusions of this article will be made available by the authors, without undue reservation.

## Ethics Statement

Ethical review and approval was not required for the study on human participants in accordance with the local legislation and institutional requirements. Written informed consent for participation was not required for this study in accordance with the national legislation and the institutional requirements.

## Author Contributions

FS has contributed to writing the introduction and literature review. RN has also contributed in the write-up of the literature review. SN has contributed in the methodology and analysis. HG wrote the discussion and theoretical and practical implications. All authors contributed to the article and approved the submitted version.

## Conflict of Interest

The authors declare that the research was conducted in the absence of any commercial or financial relationships that could be construed as a potential conflict of interest.

## Publisher’s Note

All claims expressed in this article are solely those of the authors and do not necessarily represent those of their affiliated organizations, or those of the publisher, the editors and the reviewers. Any product that may be evaluated in this article, or claim that may be made by its manufacturer, is not guaranteed or endorsed by the publisher.
